# Differences in Self-Reported and Billed Postpartum Visits Among Medicaid-Insured Individuals

**DOI:** 10.1001/jamanetworkopen.2023.49457

**Published:** 2023-12-27

**Authors:** Meghan Bellerose, Jamie R. Daw, Maria W. Steenland

**Affiliations:** 1 Department of Health Services, Policy, and Practice, Brown University School of Public Health, Providence, Rhode Island; 2 Department of Health Policy and Management, Columbia University Mailman School of Public Health, New York, New York; 3 Brown University Population Studies and Training Center, Providence, Rhode Island

## Abstract

**Importance:**

State Medicaid programs have recently implemented several policies to improve access to health care during the postpartum period. Understanding whether these policies are succeeding will require accurate measurement of postpartum visit use over time and across states; however, current estimates of use vary substantially between data sources.

**Objectives:**

To examine disagreement between postpartum visit use reported in the Pregnancy Risk Assessment Monitoring System (PRAMS) and Medicaid claims and assess whether insurance transitions from Medicaid at the time of childbirth to other insurance types after delivery are associated with the degree of disagreement.

**Design, Setting, and Participants:**

This cross-sectional study was conducted among individuals in South Carolina after delivery who had completed a PRAMS survey and for whom Medicaid was the payer of their delivery care. PRAMS responses from 2017 to 2020 were linked to inpatient, outpatient, and physician Medicaid claims; survey-weighted logistic regression models were then used to examine the association between postpartum insurance transitions and data source disagreement. Data were analyzed from February through October 2023.

**Exposure:**

Insurance transition type: continuous Medicaid, Medicaid to private insurance, Medicaid to no insurance, and Emergency Medicaid to no insurance.

**Main Outcome and Measure:**

Data source disagreement due to reporting a postpartum visit in PRAMS without a Medicaid claim for a visit or having a Medicaid claim for a visit without reporting a postpartum visit in PRAMS.

**Results:**

Among 836 PRAMS respondents enrolled in Medicaid at delivery (663 aged 20-34 years [82.9%]), a mean of 85.7% (95% CI, 82.1%-88.7%) reported a postpartum visit in PRAMS and a mean of 61.6% (95% CI, 56.9%-66.0%) had a Medicaid claim for a postpartum visit. Overall, 253 respondents (30.3%; 95% CI, 26.1%-34.7%) had data source disagreement: 230 individuals (27.2%; 95% CI, 23.2%-31.5%) had a visit in PRAMS without a Medicaid claim, and 23 individuals (3.1%; 95% CI, 1.8%-5.2%) had a Medicaid claim without a visit in PRAMS. Compared with individuals continuously enrolled in Medicaid, those who transitioned to private insurance after delivery and those who were uninsured after delivery and had Emergency Medicaid at delivery had an increase in the probability of data source agreement of 15.8 percentage points (95% CI, 2.6-29.1 percentage points) and 37.2 percentage points (95% CI, 19.6-54.8 percentage points), respectively.

**Conclusions and Relevance:**

This study’s findings suggest that Medicaid claims may undercount postpartum visits among people who lose Medicaid or switch to private insurance after childbirth. Accounting for these insurance transitions may be associated with better claims-based estimates of postpartum care.

## Key Points

**Question:**

Do transitions in health insurance coverage between delivery and the postpartum period contribute to differences in self-reported and claims-based estimates of postpartum care use?

**Findings:**

In this cross-sectional study of 836 individuals in the postpartum period, 27.2% of respondents with Medicaid at delivery self-reported a postpartum visit but did not have a Medicaid claim for a visit. The greatest differences were among people who transitioned from Emergency Medicaid at delivery to no insurance after delivery and Medicaid at delivery to private insurance after delivery.

**Meaning:**

These findings suggest that estimates of postpartum care use that are used to inform state Medicaid policy decisions could be strengthened by accounting for insurance transitions after delivery.

## Teaser

This cross-sectional study investigates the association of transitions in health care coverage with disagreement in postpartum visit use in self-reports and Medicaid claims.

## Introduction

In the US, approximately half of pregnancy-related deaths occur more than 1 week after delivery.^1^ Improving access to care during the postpartum period is a key component of the US national agenda to improve maternal health.^2^ During routine postpartum visits, clinicians conduct a full assessment of physical and psychological well-being, address urgent health care needs, and coordinate a transition to primary care for patients with chronic conditions.^3^ Since 2018, the American College of Obstetrics and Gynecology has recommended that all birthing people have initial contact with a clinician within 3 weeks of delivery and a comprehensive postpartum visit within 3 months; however, in practice, a single routine postpartum visit is typically scheduled between 4 and 6 weeks after delivery.^3^ In recognition of the importance of routine postpartum visits, receipt of such a visit is included in the Healthcare Effectiveness Data and Information Set quality measures, and national progress toward 100% attendance is reported annually.^4^

State Medicaid programs have been at the forefront of efforts to increase postpartum care access among individuals with lower incomes, who experience higher rates of severe maternal morbidity.^5,6^ These efforts include recent policy changes to expand pregnancy Medicaid eligibility and reduce immigration-related barriers to Medicaid coverage during the postpartum period.^6^ In addition, as of November 2023, 39 state Medicaid programs had adopted the American Rescue Plan Act option to extend pregnancy Medicaid coverage from conception through 1 year after delivery (from the previous 60 days).^7^ Determining whether these policies are succeeding in increasing postpartum care use will require accurate measurement between states and over time; however, estimates of postpartum visit use differ by a large margin between data sources, and little research has been conducted to reconcile differences in estimates.^8^

There are 2 primary data sources used to measure postpartum visit receipt among Medicaid-insured individuals: the Pregnancy Risk Assessment Monitoring System (PRAMS) and Medicaid claims. PRAMS is a survey conducted annually by the Centers for Disease Control and Prevention and state health departments to collect information on experiences before, during, and after pregnancy from state-representative samples of people with a live birth. Medicaid claims include fee-for-service and managed-care billing records for health care paid in part or full by Medicaid. Neither Medicaid claims nor PRAMS responses are a criterion standard for measuring postpartum care. Between 2016 and 2019, 88.8% of people with Medicaid after delivery reported attending a postpartum visit in PRAMS,^9^ while estimates based on Medicaid claims indicated that 66.7% of Medicaid enrollees received a postpartum visit.^4^

Medicaid claims may undercount postpartum visit use if people with a Medicaid-reimbursed delivery receive postpartum care that is not reimbursed by Medicaid. This could occur if a person who had delivered received care at a safety net clinic or paid for the visit out of pocket. For example, among people who are undocumented and immigrants who are within 5 years of establishing permanent residency, childbirth is often financed by Emergency Medicaid, which covers labor and delivery care for individuals who do not qualify for full-coverage Medicaid due to their immigration status.^10^ Given that Emergency Medicaid does not pay for postpartum care, any postpartum health care received by this group of immigrants would not be observable in Medicaid claims. Postpartum care paid by private insurance would also be absent from Medicaid claims. Switching to private insurance during the postpartum period after a Medicaid-paid delivery is common, occurring after 12% to 25% of Medicaid-paid births.^11,12^ Conversely, PRAMS may produce an overcount of postpartum visit attendance if people who have delivered misreport other preventive care as a postpartum visit on surveys.

A previous study^13^ compared Medicaid claims for postpartum visits with self-reported receipt of postpartum care in the 2011 to 2015 PRAMS survey in Wisconsin and found that agreement between data sources ranged from 42% to 82% depending on how postpartum care was identified in Medicaid claims. Our study extends that work by examining the association between insurance transitions and differences between postpartum visit receipt in PRAMS and Medicaid claims in South Carolina. We assessed whether insurance transitions from Medicaid at the time of childbirth to other insurance types during the postpartum period were associated with the degree of disagreement between data sources.

## Methods

This cross-sectional study followed the Strengthening the Reporting of Observational Studies in Epidemiology (STROBE) reporting guideline. The study was deemed exempt from review and informed consent by Brown University Institutional Review Boards because secondary, deidentified data were used.

### Data Sources

This study used South Carolina PRAMS survey data and Medicaid claims and enrollment files from 2017 to 2020. The South Carolina Revenue and Fiscal Affairs Office provided Medicaid enrollment files for all individuals who were mailed a PRAMS survey in this period; inpatient, outpatient, and physician claims for any Medicaid-paid care those individuals received in the year after childbirth; and an individual ID to link data sources.

### Study Population

Most previous studies that used Medicaid claims to examine receipt of postpartum care included all people whose delivery was paid for by Medicaid.^8^ Therefore, to generate a study population comparable with those in previous literature, we first restricted our sample to PRAMS respondents (2041 individuals) who were enrolled in Medicaid on the date of their delivery based on Medicaid enrollment data (1189 individuals). Given that people may be enrolled in multiple insurance types simultaneously at delivery, we further restricted our study population to people for whom Medicaid was identified as the principal payer of delivery care on the child’s birth certificate (851 individuals). For individuals with multiple PRAMS responses within the study period (15 individuals), we included only the birth corresponding to the first PRAMS response (836 individuals). A flow diagram of study inclusion is shown in the eFigure in Supplement 1.

### Outcomes

We measured routine postpartum visit attendance in PRAMS using the survey question “Since your new baby was born, have you had a postpartum checkup for yourself? (A postpartum checkup is the regular checkup a woman has about 4-6 weeks after she gives birth).” We identified postpartum care visits in outpatient Medicaid claims using the *International Statistical Classification of Diseases and Related Health Problems, Tenth Revision* (*ICD-10*) code Z39.2, which is used in South Carolina to reimburse for postpartum visits. South Carolina is 1 of 14 states that bills for postpartum visits separately from antepartum, labor, and delivery care rather than grouping payment for these services into a single perinatal care bundle billed at the time of delivery.^14^ Between 2017 and 2020, fewer than 0.5% of postpartum visits were reimbursed using bundled payment codes in South Carolina.^15^ We restricted postpartum visits in claims to those billed within 60 days after delivery to align with the postpartum care–identification strategy used by the Centers for Medicare & Medicaid Services (CMS).^16^

The study outcome was data source disagreement between PRAMS and Medicaid claims because a respondent reported a postpartum visit in PRAMS without a Medicaid claim for a visit or had a Medicaid claim for a visit without reporting a visit in PRAMS. In eTable 3 in Supplement 1, we also examined these types of data source disagreement individually.

### Demographics and Postpartum Insurance Coverage

To describe our linked PRAMS and Medicaid claims sample, we examined several demographic characteristics using self-reported information from PRAMS responses. We created a combined variable for race and ethnicity with the following groups: Hispanic, non-Hispanic Black, non-Hispanic White, and multiple races or other race not previously listed. The last category includes non-Hispanic individuals whose race was listed as American Indian, Alaska Native, Chinese, Japanese, Filipino, Hawaiian, mixed race, and other race. We collapsed these responses into 1 category owing to small sample sizes. Race responses were not assessed for people who reported Hispanic ethnicity. We also examined educational attainment (no high school diploma, high school diploma, and some college or higher) and age group (≤19, 20-24, 25-29, 30-34, and ≥35 years).

To examine whether insurance transitions from Medicaid at childbirth to another insurance type after delivery were associated with data source disagreement, we created a variable identifying respondent self-reported insurance type at the time of the PRAMS survey. This survey is completed at a median (IQR) of 4 (3-5) months after delivery.^17,18,19^ Responses were classified hierarchically into 1 of 3 mutually exclusive categories: private insurance, Medicaid, or no insurance. We also used Medicaid enrollment data to identify people whose childbirth was covered by Emergency Medicaid. We then classified respondents into 1 of 4 insurance transition types: Medicaid at delivery to Medicaid after delivery (continuous Medicaid), Medicaid at delivery to private insurance after delivery (Medicaid to private insurance), Medicaid at delivery to no insurance after delivery (Medicaid to no insurance), and Emergency Medicaid at delivery to no insurance after delivery (Emergency Medicaid to no insurance).

### Statistical Analysis

We examined differences in the prevalence of postpartum visit use between data sources by calculating survey-weighted proportions of people with a postpartum visit in PRAMS and Medicaid claims overall and by insurance transition type. We performed 2-sided *t* tests for differences in proportions. Then, we calculated the percentage of individuals with data source disagreement in the overall sample and by insurance transition type. Finally, we used survey-weighted logistic regression models to examine the association between each insurance transition type and data source disagreement. We present results as marginal effects, which can be interpreted as the percentage point difference in data source disagreement between individuals with continuous Medicaid (the reference group) and those with each insurance transition type.

Some prior studies using Medicaid claims to measure postpartum care restricted their study populations to people continuously enrolled in Medicaid during the postpartum period.^8^ Therefore, in eTables 2 and 4 in Supplement 1, we present results among PRAMS respondents (2041 individuals) who were enrolled in Medicaid on the date of their delivery based on Medicaid enrollment data (1189 individuals) and who were continuously enrolled in Medicaid from the day of delivery through 60 days after delivery based on Medicaid enrollment data (960 individuals). In addition, we present results restricted to the years 2017 to 2019 given that the start of the COVID-19 pandemic was associated with a reduction in postpartum care in 2020 relative to previous years.^9^

We conducted analyses in Stata statistical software version 17 (StataCorp). Analyses were weighted to account for the PRAMS survey design. We set a significance level of *P* < .05 a priori. Data were analyzed from February through October 2023.

## Results

### Sample Characteristics

Our sample included 836 people (663 ages 20-34 years [82.9%; 95% CI, 79.3%-86.0%]; 363 Black non-Hispanic [44.7%; 95% CI, 40.1%-49.4%], 100 Hispanic [12.3%; 95% CI 9.7%-15.5%], and 329 White non-Hispanic [37.1%; 95% CI, 32.9%-41.6%] among 834 individuals with race and ethnicity data) with a birth in South Carolina between 2017 and 2020 who completed a PRAMS survey, were enrolled in Medicaid on the date of their delivery, and for whom Medicaid was the expected payer of delivery on their child’s birth certificate (population numbers are unweighted, and percentages are weighted) (Table 1). Among respondents, one-fifth did not have a high school diploma (152 individuals [20.5%; 95% CI, 16.9%-24.6%]). Among 809 respondents who provided information on insurance at the time of the PRAMS survey, 549 individuals (67.7%; 95% CI, 63.2%-71.8%) reported having Medicaid, 117 individuals (15.4%; 95% CI, 12.3%-19.2%) reported private insurance, and 143 individuals (16.9%; 95% CI, 13.8%-20.5%) reported no insurance, including 50 individuals (6.0%; 95% CI, 4.3%-8.3%) with and 93 individuals without (10.9%; 95% CI, 8.4%-14.1%) Emergency Medicaid at childbirth. Characteristics by insurance transition type and among individuals continuously enrolled in Medicaid through 60 days after delivery are presented in eTables 1 and 2 in Supplement 1, respectively.

**Table 1.** Study Population Demographic and Insurance Characteristics

Characteristic	Respondents, No. (%) [95% CI] (N = 836)^a^
Race and ethnicity (n = 834)	
Black non-Hispanic	363 (44.7) [40.1-49.4]
Hispanic	100 (12.3) [9.7-15.5]
White non-Hispanic	329 (37.1) [32.9-41.6]
Multiple races or not previously listed^b^	42 (5.9) [4.1-8.5]
Education	
No high school diploma	152 (20.5) [16.9-24.6]
High school diploma	286 (38.4) [34.0-43.1]
Some college or higher	398 (41.1) [36.7-45.6]
Age, y	
<19	58 (7.8) [5.5-10.8]
20-24	219 (27.1) [23.2-31.5]
25-29	264 (33.8) [29.6-38.3]
30-34	180 (22.0) [18.4-26.0]
≥35	115 (9.3) [7.2-12.0]
Insurance transition between delivery and PRAMS survey (n = 809)	
Continuous Medicaid	549 (67.7) [63.2-71.8]
Medicaid to private insurance	117 (15.4) [12.3-19.2]
Medicaid to no insurance	93 (10.9) [8.4-14.1]
Emergency Medicaid to no insurance	50 (6.0) [4.3-8.3]

Abbreviation: PRAMS, Pregnancy Risk Assessment Monitoring System.

^a^ Numbers are unweighted, and percentages are survey weighted.

^b^ This category includes non-Hispanic individuals whose race was listed as American Indian, Alaska Native, Chinese, Japanese, Filipino, Hawaiian, mixed race, and other race.

### Prevalence of Postpartum Visit Use

Within our sample, a mean of 85.7% (95% CI, 82.1%-88.7%) of respondents reported a postpartum visit in PRAMS and a mean of 61.6% (95% CI, 56.9%-66.0%) had a Medicaid claim for a visit (Table 2). Differences in prevalence between data sources were greatest among individuals with a transition from Emergency Medicaid at childbirth to no insurance after delivery; a mean of 81.9% (95% CI, 63.8%-92.0%) reported a postpartum visit in PRAMS, and a mean of 19.0% (95% CI, 7.8%-39.5%) had a Medicaid claim for a visit (2-sample *t* test *P* < .001). There were also significant differences in prevalence between data sources among individuals with a Medicaid to private insurance transition and those with continuous Medicaid. Among respondents with a Medicaid to private transition, a mean of 90.6% (95% CI, 80.2%-95.8%) reported a postpartum visit in PRAMS and a mean of 53.8% (95% CI, 41.2%-65.9%) had a Medicaid claim for a visit (*P* < .001). Among respondents with continuous Medicaid, a mean of 84.7% (95% CI, 80.0%-88.5%) reported a postpartum visit in PRAMS and a mean of 64.0% (95% CI, 58.2%-69.5%) had a Medicaid claim (*P* < .001). There was not a significant difference in prevalence among individuals with a Medicaid to no insurance transition; a mean of 85.6% (95% CI, 72.2%-93.1%) reported a visit in PRAMS, and a mean of 77.9% (95% CI, 64.5%-87.2%) had a claim (*P* = .39).

**Table 2.** Prevalence of Postpartum Visit Use

Postpartum insurance transition type	Respondents with postpartum visit, mean (95% CI), %		2-Sided *t* test	
	Self-reported on PRAMS	Medicaid claim	Risk difference (95% CI), %	*P* value
Overall	85.7 (82.1-88.7)	61.6 (56.9-66.0)	19.1 (14.6-23.6)	<.001
Continuous Medicaid	84.7 (80.0-88.5)	64.0 (58.2-69.5)	15.6 (10.4-20.7)	<.001
Medicaid to private insurance	90.6 (80.2-95.8)	53.8 (41.2-65.9)	29.5 (15.3-43.7)	<.001
Medicaid to no insurance	85.6 (72.2-93.1)	77.9 (64.5-87.2)	6.1 (−8.0 to 20.2)	.39
Emergency Medicaid to no insurance	81.9 (63.8-92.0)	19.0 (7.8-39.5)	64.5 (45.6-83.5)	<.001

Abbreviation: PRAMS, Pregnancy Risk Assessment Monitoring System.

### Data Source Disagreement

Among 773 respondents with PRAMS postpartum insurance and attendance responses, 253 respondents (30.3%; 95% CI, 26.1%-34.7%) in our sample had data source disagreement, including 230 individuals (27.2%; 95% CI, 23.2%-31.5%) who reported a visit in PRAMS but did not have a Medicaid claim for a visit and 23 individuals (3.1%; 95% CI, 1.8%-5.2%) who had a Medicaid claim but did not report a visit in PRAMS (Figure; Table 3). The percentage of respondents with data source disagreement was 62.9% (95% CI, 43.5%-78.8%), 41.5% (30.1%-53.9%), 25.7% (95% CI, 20.9%-31.1%), and 22.8% (95% CI, 12.8%-37.2%) in the Emergency Medicaid to no insurance, Medicaid to private insurance, continuous Medicaid, and Medicaid to no insurance transition groups, respectively.

**Figure.**
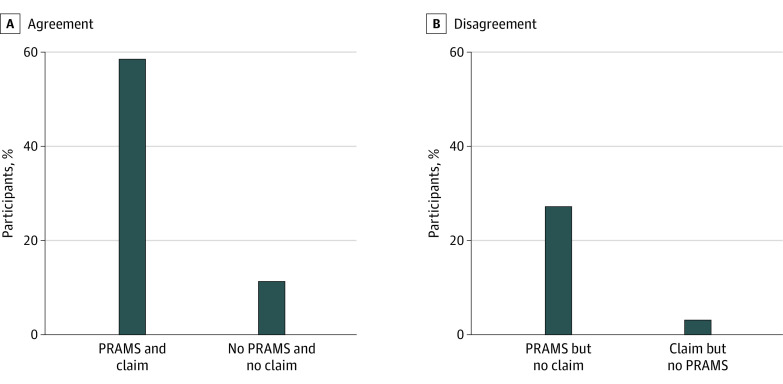
Data Source Agreement on Postpartum Visits Agreement is displayed for self-reported postpartum visit attendance in the Pregnancy Risk Assessment Monitoring System (PRAMS) and postpartum visits in Medicaid claims.

**Table 3.** Data Source Disagreement by Postpartum Insurance Transition

Postpartum insurance transition type	Respondents with postpartum visit, No. (%) [95% CI] (N = 773)^a^				Any disagreement, No. (%) [95% CI]	Difference in any disagreement, (95% CI), percentage points	*P* value
	Yes PRAMS		No PRAMS				
	Yes claim (n = 425)	No claim (n = 230)	No claim (n = 95)	Yes claim (n = 23)			
Continuous Medicaid	306 (61.5) [55.7-67.1]	141 (23.2) [18.6-28.5]	65 (12.8) [9.3-17.3]	17 (2.5) [1.2-5.0]	158 (25.7) [20.9-31.1]	0 [Reference]	NA
Medicaid to private insurance	50 (51.4) [39.2-63.5]	47 (39.1) [27.8-51.8]	9 (7.1) [2.9-16.5]	2 (2.3) [0.4-19.8]	49 (41.5) [30.1-53.9]	15.8 (2.6 to 29.1)	.02
Medicaid to no insurance	62 (70.3) [56.3-81.4]	14 (15.3) [7.5-28.5]	9 (6.9) [2.6-17.2]	4 (7.5) [2.4-21.6]	18 (22.8) [12.8-37.2]	−2.9 (−15.9 to 10.1)	.67
Emergency Medicaid to no insurance	7 (19.0) [7.8-39.5]	28 (62.9) [43.5-78.8]	12 (18.1) [8.0-36.2]	0	28 (62.9) [43.5-78.8]	37.2 (19.6 to 54.8)	<.001

Abbreviations: NA, not applicable; PRAMS, Pregnancy Risk Assessment Monitoring System.

^a^ Numbers are unweighted, and percentages are survey weighted. A total of 63 people did not respond to the PRAMS question on postpartum insurance type or attendance and were excluded from these results.

Respondents with a transition from Emergency Medicaid to no insurance had an increase in the probability of data source disagreement of 37.2 percentage points (95% CI, 19.6-54.8 percentage points) compared with respondents with continuous Medicaid. Respondents with a transition from Medicaid to private insurance had an increase in the probability of data source disagreement of 15.8 percentage points (95% CI, 2.6-29.1 percentage points) compared with respondents with continuous Medicaid. The probability of data source disagreement among respondents with a transition from Medicaid to no insurance was not statistically significantly different compared with respondents with continuous Medicaid.

Results stratified by type of data source disagreement are presented in eTable 3 in Supplement 1, along with differences in the probability of data source disagreement within each demographic group compared with a reference group. Coefficients were similar in magnitude and statistical significance when the regression outcome was a self-reported visit in PRAMS without a Medicaid claim and among people with continuous Medicaid enrollment through 60 days after delivery (eTable 4 in Supplement 1). Results excluding 2020 data are presented in eTables 5 and 6 in Supplement 1. The prevalence of data source disagreement and the model coefficients were similar in magnitude and significance, with the exception that respondents with a transition from Medicaid to private insurance no longer had a significantly higher probability of data source disagreement compared with those with continuous Medicaid.

## Discussion

In this cross-sectional study comparing postpartum visit receipt between South Carolina PRAMS and Medicaid claims, we found a 69.7% agreement rate between data sources, with more than one-quarter of our sample reporting that they attended a postpartum visit in PRAMS without a Medicaid claim for a visit. Having a transition from Medicaid at childbirth to private insurance after delivery and being uninsured postpartum after having Emergency Medicaid as payer of childbirth were associated with a greater probability of data source disagreement compared with remaining enrolled in Medicaid after delivery.

We found that data source disagreement was greatest among people who were uninsured after childbirth if they had Emergency Medicaid at delivery. Emergency Medicaid reimburses hospitals for labor and delivery care provided to individuals who are ineligible for Medicaid due to their immigration status.^10^ However, it covers only the hospitalization for childbirth without any coverage for care in the postpartum period. This finding suggests that state-level estimates of postpartum visit use derived from Medicaid claims data may systematically underestimate postpartum visit receipt by immigrants who are undocumented and recent immigrants, who may be accessing care at safety net clinics or paying out of pocket.

We also found high rates of data source disagreement among people with a transition from Medicaid at childbirth to private insurance at the time of the PRAMS survey. In our sample, 15.4% of people with Medicaid at delivery reported private insurance at the time of the PRAMS survey. This high percentage is expected given that income eligibility limits for pregnancy Medicaid coverage in South Carolina, which had not adopted the Affordable Care Act (ACA) Medicaid expansion as of June 2023, are much higher than income eligibility limits for parental Medicaid coverage. Between 2017 and 2020, South Carolina residents with incomes less than 199% of the federal poverty level were eligible for Medicaid during pregnancy through 60 days after delivery, and parents with incomes less than 67% of the federal poverty level were eligible for Medicaid coverage thereafter.^20^ This pattern held within our secondary sample of people who were continuously enrolled in Medicaid from delivery through 60 days after delivery but who self-reported private insurance on the PRAMS survey. These individuals may have been simultaneously enrolled in Medicaid and private insurance, in which case private insurance would typically serve as the first line of coverage for routine postpartum care.^21^ In contrast, we did not find significant data source disagreement among people with a transition from Medicaid at childbirth to no insurance at the time of the PRAMS survey. It is likely that individuals in this group were able to obtain a Medicaid-reimbursed postpartum visit within the typical range of 4 to 6 weeks after delivery before their pregnancy Medicaid coverage ended at 60 days after delivery.

Notably, we found high rates of data source disagreement even for people whose delivery care was paid for by Medicaid and who remained covered by Medicaid during the postpartum period. This finding suggests that other factors beyond insurance transitions may be associated with differences in measurement between data sources. One important possibility that we did not examine in this analysis is that some respondents may misreport having received a postpartum visit in PRAMS. Individuals who have delivered may receive a variety of health care visit types during the postpartum period that may be mistaken for a comprehensive postpartum visit, such as a postpartum cesarean wound check or health care visit to address acute health concerns.^22^ To our knowledge, no studies have validated the PRAMS postpartum visit question; however, previous research has used electronic health records to test the validity of a variety of other PRAMS questions.^23,24^

This study’s findings have several important health policy implications. State-level postpartum visit use estimated from Medicaid claims is used by the CMS as part of its Medicaid and Children’s Health Insurance Program Scorecard.^25^ There is variation among states in the share of births to people who switch from Medicaid to private insurance coverage and the share of births to immigrants who are ineligible for Medicaid due to their immigration status.^11^ Our findings suggest that postpartum visit rates may be underestimated at a higher rate in states with greater shares of birthing persons who experience these 2 types of postpartum insurance transitions. This includes states that have not adopted the ACA Medicaid expansion, have large shares of undocumented birthing persons, and have not adopted policies to waive the 5-year waiting period for permanent residents.

Underestimating postpartum visit receipt due to Medicaid to private insurance transitions after delivery could also complicate efforts to track progress over time within a state. This is because major insurance policy changes, such as state Medicaid expansion, affect rates of postpartum insurance churn.^11^ Recent extensions to postpartum Medicaid through the full postpartum year may increase postpartum care receipt; however, this policy may also reduce private insurance churn, making it difficult to disentangle any real increase in postpartum visits attributable to the policy from increases in documentation of care in Medicaid claims data. Finally, people covered by Emergency Medicaid are disproportionally likely to be Hispanic. Therefore, use of Medicaid claims to measure racial and ethnic disparities in postpartum care may lead to misleading conclusions about racial and ethnic inequities in postpartum care receipt.

With the release of Transformed Medicaid Statistical Information System Analytic Files by the CMS beginning in 2019, use of Medicaid claims for research on postpartum health care use and outcomes may increase.^14^ For researchers planning to use Medicaid claims data to assess postpartum care use and outcomes, our findings suggest that measurement could be strengthened by stratifying results by Emergency Medicaid status and requiring continuous enrollment in Medicaid for the period of measurement of postpartum outcomes for individuals without Emergency Medicaid.

### Limitations

This study has several limitations. It did not have a criterion standard data source to assess the reliability of self-reported PRAMS responses and Medicaid claims independently. Therefore, we were not able to examine other potential measurement issues, most notably the degree to which respondents may have overreported postpartum visit attendance in PRAMS. Recall bias may be greater for individuals who completed a PRAMS survey further from their delivery date. Additionally, these results may not be equally generalizable to all states due to differences in Medicaid billing practices and eligibility criteria. For example, as noted previously, South Carolina is one of few states that do not bundle payment for prenatal, delivery, and postpartum care.^14^ The ability to identify postpartum visits separately from other maternity care in South Carolina claims allowed us to focus our analysis on the contribution of postpartum insurance transitions to explaining measurement discrepancies between data sources. However, in states with bundled payment codes, measurement differences between PRAMS and Medicaid claims could result from global payment billing practices, postpartum insurance transitions, and other potential factors.

## Conclusions

Medicaid claims data are a useful source of information on health care use during the postpartum period; however, results from this cross-sectional study suggest that estimates of postpartum visit use derived from Medicaid claims may undercount postpartum visit receipt by systematically missing care received by individuals with Emergency Medicaid for delivery and those who transition to private insurance during the postpartum period. These findings suggest that accounting for postpartum insurance transitions when conducting studies using Medicaid claims data may be associated with improved estimates of postpartum health care use and outcomes.

## Notes

Supplement 1. **eFigure.** Flow Diagram of Study Inclusion **eTable 1.** Study Population Demographic and Insurance Characteristics by Insurance Transition After Delivery **eTable 2.** Demographic and Insurance Characteristics of the Secondary Population of People Continuously Enrolled in Medicaid for 60 d After Delivery **eTable 3.** Factors Associated With Postpartum Visit Data Source Disagreement in Primary Study Population **eTable 4.** Data Source Disagreement by Postpartum Insurance Transition in Secondary Population of People Continuously Enrolled in Medicaid for 60 d After Delivery **eTable 5.** Prevalence of Postpartum Visit Use by Data Source Excluding 2020 Data **eTable 6.** Data Source Disagreement by Postpartum Insurance Transition Excluding 2020 Data

Supplement 2.
Data Sharing Statement

## References

[1] Trost S, Beauregard J, Chandra G, . Pregnancy-related deaths: data from maternal mortality review committees in 36 US states, 2017-2019. 2022. Accessed December 6, 2023. https://www.cdc.gov/reproductivehealth/maternal-mortality/erase-mm/data-mmrc.html

[2] US Department of Health and Human Services. Healthy women, healthy pregnancies, healthy futures: action plan to improve maternal health in America. US Department of Health and Human Services; 2020. Accessed November 17, 2023. https://health.gov/healthypeople/tools-action/browse-evidence-based-resources/healthy-women-healthy-pregnancies-healthy-futures-action-plan-improve-maternal-health-america

[3] American College of Obstetrics and Gynecologists. Optimizing postpartum care. Accessed October 20, 2021. https://www.acog.org/clinical/clinical-guidance/committee-opinion/articles/2018/05/optimizing-postpartum-care

[4] National Committee for Quality Assurance. Prenatal and postpartum care (PPC): HEDIS measures and technical resources. Accessed November 17, 2023. https://www.ncqa.org/hedis/measures/prenatal-and-postpartum-care-ppc/

[5] Howell EA, Egorova NN, Janevic T, . Race and ethnicity, medical insurance, and within-hospital severe maternal morbidity disparities. Obstet Gynecol. 2020;135(2):285-293. doi:10.1097/AOG.0000000000003667 31923076 PMC7117864

[6] Clark M, Millette M. State opportunities to leverage Medicaid and CHIP coverage to improve maternal health and eliminate racial inequities. Accessed November 17, 2023. https://ccf.georgetown.edu/2023/04/25/opportunities-to-leverage-medicaid-and-chip-to-improve-maternal-health-and-eliminate-racial-inequities/

[7] Kaiser Family Foundation. Medicaid postpartum coverage extension tracker. Accessed June 2, 2023. https://www.kff.org/medicaid/issue-brief/medicaid-postpartum-coverage-extension-tracker/

[8] Attanasio LB, Ranchoff BL, Cooper MI, Geissler KH. Postpartum visit attendance in the United States: a systematic review. Womens Health Issues. 2022;32(4):369-375. doi:10.1016/j.whi.2022.02.002 35304034 PMC9283204

[9] Bellerose M, Steenland MW. Association between the coronavirus disease 2019 (COVID-19) pandemic and national disparities in postpartum visit attendance. Obstet Gynecol. 2023;141(1):170-172. doi:10.1097/AOG.0000000000005014 36701617 PMC10829906

[10] Khullar D, Chokshi DA. Immigrant health, value-based care, and Emergency Medicaid reform. JAMA. 2019;321(10):928-929. doi:10.1001/jama.2019.0839 30860550

[11] Daw JR, Winkelman TNA, Dalton VK, Kozhimannil KB, Admon LK. Medicaid expansion improved perinatal insurance continuity for low-income women. Health Aff (Millwood). 2020;39(9):1531-1539. doi:10.1377/hlthaff.2019.01835 32897793

[12] Ela EJ, Vizcarra E, Thaxton L, White K. Insurance churn and postpartum health among Texas women with births covered by Medicaid/CHIP. Womens Health Issues. 2022;32(2):95-102. doi:10.1016/j.whi.2021.11.002 34916138 PMC8940665

[13] DeSisto CL, Rohan A, Handler A, Awadalla SS, Johnson T, Rankin K. Comparing postpartum care utilization from Medicaid claims and the Pregnancy Risk Assessment Monitoring System in Wisconsin, 2011-2015. Matern Child Health J. 2021;25(3):428-438. doi:10.1007/s10995-021-03118-2 33523347

[14] Daw JR, Auty SG, Admon LK, Gordon SH. Using modernized Medicaid data to advance evidence-based improvements in maternal health. Am J Public Health. 2023;113(7):805-810. doi:10.2105/AJPH.2023.307287 37141557 PMC10262233

[15] Centers for Medicare and Medicaid Services. Perinatal care services provided to Medicaid and CHIP beneficiaries ages 15 to 44. Accessed November 17, 2023. https://data.medicaid.gov/dataset/ed67e610-aed3-4bed-842f-e6044511dd64

[16] Centers for Medicare and Medicaid Services. Quality ID #336: maternity care: postpartum follow-up and care coordination. Accessed November 17, 2023. https://qpp.cms.gov/docs/QPP_quality_measure_specifications/CQM-Measures/2020_Measure_336_MIPSCQM.pdf

[17] Daw JR, Kozhimannil KB, Admon LK. Factors associated with postpartum uninsurance among Medicaid-paid births. JAMA Health Forum. 2021;2(6):e211054. doi:10.1001/jamahealthforum.2021.1054 35977176 PMC8796978

[18] Eliason E, Admon LK, Steenland MW, Daw JR. Late postpartum coverage loss before COVID-19: implications for Medicaid unwinding. Health Aff (Millwood). 2023;42(7):966-972. doi:10.1377/hlthaff.2022.01659 37406233 PMC10885010

[19] Luff A, Menegay M, Gallo MF. Prevalence and correlates of very rapid repeat pregnancy: Pregnancy Risk Assessment Monitoring System, United States, 2009-2020. Paediatr Perinat Epidemiol. Published online October 23, 2023. doi:10.1111/ppe.13014 37872870 PMC10841439

[20] Kaiser Family Foundation. Medicaid Income Eligibility Limits for Parents, 2002-2023. Accessed November 17, 2023. https://www.kff.org/medicaid/state-indicator/medicaid-income-eligibility-limits-for-parents/?currentTimeframe=0&sortModel=%7B%22colId%22:%22Location%22,%22sort%22:%22asc%22%7D

[21] Centers for Medicare and Medicaid Services. How Medicare works with other insurance. Accessed December 6, 2023. https://www.medicare.gov/supplements-other-insurance/how-medicare-works-with-other-insurance

[22] Steenland MW, Kozhimannil KB, Werner EF, Daw JR. Health care use by commercially insured postpartum and nonpostpartum women in the United States. Obstet Gynecol. 2021;137(5):782-790. doi:10.1097/AOG.0000000000004359 33831924 PMC8058261

[23] Ahluwalia IB, Helms K, Morrow B. Assessing the validity and reliability of three indicators self-reported on the pregnancy risk assessment monitoring system survey. Public Health Rep. 2013;128(6):527-536. doi:10.1177/003335491312800612 24179264 PMC3804096

[24] Dietz P, Bombard J, Mulready-Ward C, . Validation of self-reported maternal and infant health indicators in the Pregnancy Risk Assessment Monitoring System. Matern Child Health J. 2014;18(10):2489-2498. doi:10.1007/s10995-014-1487-y 24770954 PMC4560102

[25] Centers for Medicare and Medicaid Services. Quality of maternal and perinatal health care in Medicaid and CHIP: findings from the 2020 Maternity Core Set. 2021. Accessed December 6, 2023. https://www.medicaid.gov/sites/default/files/2021-11/2021-maternity-chart-pack.pdf

