# Supplementary material for: Differences in Self-Reported and Billed Postpartum Visits Among Medicaid-Insured Individuals
Source: JAMA Netw Open. 2023 Dec 27;6(12):e2349457. doi: 10.1001/jamanetworkopen.2023.49457 (PMC10753392; doi:10.1001/jamanetworkopen.2023.49457)
Supplement: Supplement 1. — eFigure. Flow Diagram of Study Inclusion eTable 1. Study Population Demographic and Insurance Characteristics by Insurance Transition After Delivery eTable 2. Demographic and Insurance Characteristics of the Secondary Population of People Continuously Enrolled in Medicaid for 60 d After Delivery eTable 3. Factors Associated With Postpartum Visit Data Source Disagreement in Primary Study Population eTable 4. Data Source Disagreement by Postpartum Insurance Transition in Secondary Population of People Continuously Enrolled in Medicaid for 60 d After Delivery eTable 5. Prevalence of Postpartum Visit Use by Data Source Excluding 2020 Data eTable 6. Data Source Disagreement by Postpartum Insurance Transition Excluding 2020 Data [file jamanetwopen-e2349457-s001.pdf]

Supplemental Online Content

Bellerose M, Daw JR, Steenland MW. Differences in self-reported and billed postpartum visits among Medicaid-insured women. *JAMA Netw Open*. 2023;6(12):e2349457. doi:10.1001/jamanetworkopen.2023.49457

eFigure. Flow Diagram of Study Inclusion
eTable 1. Study Population Demographic and Insurance Characteristics by Insurance Transition After Delivery
eTable 2. Demographic and Insurance Characteristics of the Secondary Population of People Continuously Enrolled in Medicaid for 60 d After Delivery
eTable 3. Factors Associated With Postpartum Visit Data Source Disagreement in Primary Study Population
eTable 4. Data Source Disagreement by Postpartum Insurance Transition in Secondary Population of People Continuously Enrolled in Medicaid for 60 d After Delivery
eTable 5. Prevalence of Postpartum Visit Use by Data Source Excluding 2020 Data
eTable 6. Data Source Disagreement by Postpartum Insurance Transition Excluding 2020 Data

This supplemental material has been provided by the authors to give readers additional information about their work.

© 2023 Bellerose M et al. *JAMA Network Open.*

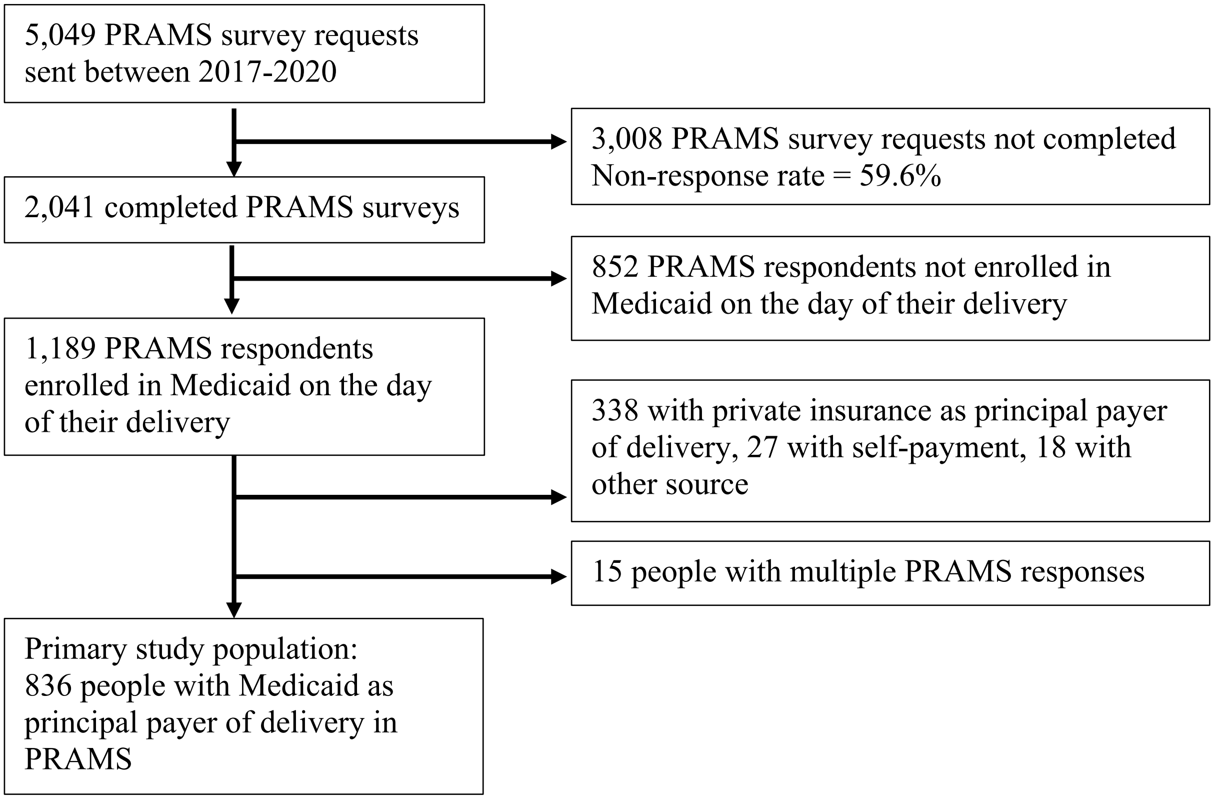

eFigure 1. Flow Diagram of Study Inclusion

© 2023 Bellerose M et al. *JAMA Network Open.*

eTable 1. Study Population Demographic and Insurance Characteristics by Postpartum Insurance Transition

	Continuous Medicaid			Medicaid to Private			Medicaid to No Insurance			Emergency Medicaid to No Insurance		
	N[a]	%	95% CI	N	%	95% CI	N	%	95% CI	N	%	95% CI
Total	549	67.7	63.2, 71.8	117	15.4	12.3, 19.2	93	10.9	8.4, 14.1	50	6.0	4.3, 8.3
Race / ethnicity												
Black NH	259	48.7	42.9, 54.5	63	56.2	43.7, 68.0	31	28.2	17.6, 41.9	1	2.2	0.3, 15.3
Hispanic	31	6.4	4.1, 9.8	13	11.0	5.4, 21.1	11	13.0	6.4, 24.4	43	87.6	70.2, 95.5
White NH	234	38.5	33.2, 44.1	36	20.4	20.7, 42.1	44	53.1	38.9, 66.8	2	4.3	0.6, 24.5
Other / Mixed Race NH	24	6.5	4.1, 10.0	4	2.5	0.5, 11.9	7	5.8	1.7, 17.8	4	6.0	1.5, 21.7
Education												
No high school diploma	95	18.4	14.3, 23.5	17	18.7	10.5, 30.9	14	23.0	12.6, 38.3	22	43.3	26.4, 62.2
High school diploma	203	43.3	37.6, 49.2	30	33.2	22.4, 46.1	27	23.9	13.7, 38.3	18	33.6	19.8, 50.9
Some college or higher	251	38.3	33.0, 43.9	70	48.2	36.0, 60.5	52	53.1	39.1, 66.6	10	23.0	11.3, 41.1
Age (years)												
Under 19	42	8.4	5.6, 12.4	7	3.3	9.1, 11.0	5	11.3	4.4, 25.8	2	2.8	0.5, 15.8
20-24	137	26.4	21.5, 31.9	39	39.5	27.9, 52.5	27	27.0	16.7, 40.6	11	13.2	5.3, 29.2
25-29	175	33.5	28.2, 29.3	39	31.9	21.7, 44.3	28	36.8	24.4, 51.2	15	34.6	19.6, 53.5
30-34	118	23.1	18.5, 28.4	21	15.9	9.2, 26.0	20	14.1	7.4, 25.0	14	37.9	21.3, 57.3
35 or older	77	8.7	6.2, 12.0	11	9.4	4.7, 18.1	13	10.8	4.7, 23.0	8	11.5	4.3, 27.1

[a] Ns are unweighted and percentages are survey weighted

© 2023 Bellerose M et al. *JAMA Network Open.*

eTable 2. Demographic and Insurance Characteristics of the Secondary Population of People Continuously Enrolled in Medicaid for 60 Days Postpartum

	N[a]	%	95% CI
Total	960	100	--
Race / ethnicity			
Black NH	417	43.9	39.6, 48.3
Hispanic	65	7.6	5.7, 10.1
White NH	435	43.6	39.5, 47.9
Other / Mixed Race NH	40	4.9	3.3, 7.1
Education			
No high school diploma	130	16.2	13.1, 19.8
High school diploma	298	35.1	31.0, 39.5
Some college or higher	532	48.7	44.4, 53.0
Age (years)			
Under 19	56	6.4	4.6, 8.9
20-24	248	28.5	24.7, 32.7
25-29	311	34.7	30.7, 39.0
30-34	202	19.9	16.8, 23.5
35 or older	143	10.5	8.3, 13.14
Insurance transition between delivery and PRAMS survey			
Continuous Medicaid	559	61.6	57.2, 65.7
Medicaid to Private	255	26.1	22.5, 30.1
Medicaid to No Insurance	111	12.3	9.8, 15.5
Emergency Medicaid to No Insurance	--	--	--

[a] Ns are unweighted and percentages are survey weighted

© 2023 Bellerose M et al. *JAMA Network Open.*

eTable 3. Predictors of Postpartum Visit Data Source Disagreement in the Primary Study Population

	Difference in disagreement (95% CI), percentage points		
	Any disagreement	Yes PRAMS / No claim disagreement	No PRAMS / Yes claim disagreement
Race / ethnicity			
Non-Hispanic Black	8.5 (-0.8, 17.8)	7.1 (-1.8, 16.1)	1.4 (-2.1, 4.9)
Hispanic	21.0 (7.0, 34,9)**	18.3 (4.7, 31.9)**	2.7 (-3.8, 9.1)
Non-Hispanic White (ref)			
Non-Hispanic other / multiple	-4.1 (-19.0, 10.8)	-2.4 (-17.2, 12.3)	-1.7 (-4.2, 0.8)
Education			
No high school diploma	9.6 (-2.3, 21.6)	2.7 (-8.4, 13.8)	6.9 (0.3, 13.5)*
High school diploma	3.7 (-5.7, 13.1)	4.5 (-4.7, 13.7)	-0.7 (-3.3, 1.8)
Some college or more (ref)			
Age (years)			
Under 19	-0.6 (-16.9, 15.7)	-2.9 (-18.2, 12.4)	2.3 (-4.6, 9.1)
20-24	7.5 (-4.2, 19.2)	6.4 (-4.9, 17.7)	1.0 (-3.2, 5.3)
25-29	9.2 (-1.8, 20.2)	7.0 (-3.6, 17.6)	2.2 (-2.1, 6.5)
30-34 (ref)			
35 or older	14.1 (-1.1, 29.3)	13.1 (-1.7, 28.0)	0.9 (-4.4, 6.2)
Insurance transition between delivery and PRAMS survey			
Continuous Medicaid	0 [Reference]	0 [Reference]	0 [Reference]
Medicaid to Private	15.8 (2.6, 29.1)*	16.0 (2.9, 29.0)*	-0.1 (-4.7, 4.4)
Medicaid to No Insurance	-2.9 (-15.9, 10.1)	-7.9 (-19.0, 3.2)	5.0 (-3.3, 13.4)
Emergency Medicaid to No Insurance	37.2 (19.6, 54.8)***	39.7 (22.1, 57.3)***	-2.5 (-4.2, -0.8)**

*** p value <0.001, ** p value <0.01, * p value <0.05

© 2023 Bellerose M et al. *JAMA Network Open.*

eTable 4. Data Source Disagreement by Postpartum Insurance Transition in Secondary Population of People Continuously Enrolled in Medicaid for 60 Days Postpartum

	Respondents with postpartum visit, No. [% (95% CI)] (N=920)[a]				Any disagreement N (%) [95% CI]	Difference in ant disagreement (95% CI), percentage points	*P* value
	Yes PRAMS		No PRAMS				
	Yes Claim (n=489)	No Claim (n=329)	No Claim (n=79)	Yes Claim (n=23)			
Continuous Medicaid	318 (63.0) [57.1, 68.4]	147 (23.0) [18.5, 28.2]	59 (12.1) [8.7, 16.5]	16 (2.0) [0.9, 4.1]	163 (25.0) [20.3, 20.3]	0 [Reference]	NA
Medicaid to Private	86 (45.1) [36.7, 53.8]	146 (51.5) [43.0, 60.0]	8 (3.0) [1.2, 7.1]	1 (0.4) [0, 2.8]	147 (51.9) [43.4, 60.4]	27.0 (17.1, 36.9)	<0.001
Medicaid to No Insurance	71 (62.4) [49.1, 74.1]	21 (21.0) [12.2, 33.6]	9 (8.2) [3.6, 17.9]	5 (8.4) [3.1, 21.1]	26 (29.4) [18.7, 42.9]	4.4 (-8.3, 17.2)	0.50
Emergency Medicaid to No Insurance	NA	NA	NA	NA	NA	NA	NA

Abbreviations: NA, not applicable; PRAMS, Pregnancy Risk Assessment Monitoring System

[a] Ns are unweighted and percentages are survey weighted

[b] A total of 40 people did not respond to the PRAMS question on postpartum insurance type and were excluded from these results

© 2023 Bellerose M et al. *JAMA Network Open.*

eTable 5. Prevalence of Postpartum Visit Use by Data Source, Excluding 2020 Data

Postpartum insurance transition type	Respondents with postpartum visit, mean % (95% CI)		2-sided *t* test	
	Self-reported on PRAMS<br><br>Mean (95% CI)	Medicaid claim<br><br>Mean (95% CI)	Risk difference, percentage	*P* value
Overall	86.8 (82.8, 90.0)	63.7 (58.3, 68.8)	17.7 (12.7, 22.6)	< 0.001
Continuous Medicaid	86.1 (80.7, 90.1)	64.5 (57.6, 70.8)	15.1 (9.2, 21.1)	< 0.001
Medicaid to Private	92.8 (82.3, 97.3)	61.4 (45.7, 75.1)	26.1 (11.7, 40.4)	.01
Medicaid to No Insurance	84.0 (69.4, 92.4)	75.8 (61.2, 86.2)	6.4 (-9.2, 22.0)	.41
Emergency Medicaid to No Insurance	80.5 (54.3, 93.9)	25.4 (9.0, 53.9)	54.7 (28.4, 81.0)	<0.001

© 2023 Bellerose M et al. *JAMA Network Open.*

eTable 6. Data Source Disagreement by Postpartum Insurance Transition, Excluding 2020 Data

	Respondents with postpartum visit, No. (%) [95% CI] (N=978)[a]				Any disagreement N (%) [95% CI]	Difference in ant disagreement (95% CI), percentage points	*P* value
	Yes PRAMS		No PRAMS				
	Yes Claim (n=341)	No Claim (n=159)	No Claim (n=72)	Yes Claim (n=17)			
Continuous Medicaid	226 (62.4) [55.5, 68.8]	94 (23.6) [18.3, 30.0]	48 (11.9) [8.1, 17.0]	12 (2.1) [0.9, 4.8]	106 (25.7) [20.2, 32.2]	0 [Reference]	NA
Medicaid to Private	40 (61.1) [45.4, 74.8]	32 (31.7) [19.3, 47.4]	7 (6.9) [2.5, 17.6]	1 (0.3) [0, 2.1]	33 (32.0) [19.5, 47.7]	6.2 (-8.9, 21.4)	.42
Medicaid to No Insurance	56 (67.5) [52.2, 79.8]	13 (16.6) [8.0, 31.1]	9 (7.6) [2.8, 18.9]	4 (8.3) [2.6, 23.7]	17 (24.9) [13.9, 40.6]	-0.8 (-15.1, 13.5)	.91
Emergency Medicaid to No Insurance	6 (25.4) [9.0, 53.9]	14 (55.1) [29.8, 78.0]	7 (19.5) [6.5, 45.7]	0 (0)	14 (55.1) [29.8, 78.0]	29.4 (5.9, 52.9)	.01

Abbreviations: NA, not applicable; PRAMS, Pregnancy Risk Assessment Monitoring System

[a] Ns are unweighted; percentages are survey weighted

[b] A total of 59 people did not respond to the PRAMS question on postpartum insurance type and were excluded from these results

© 2023 Bellerose M et al. *JAMA Network Open.*
