# Supplementary material for: Differences in Self-Reported and Billed Postpartum Visits Among Medicaid-Insured Individuals
Source: JAMA Netw Open. 2023 Dec 27;6(12):e2349457. doi: 10.1001/jamanetworkopen.2023.49457 (PMC10753392; doi:10.1001/jamanetworkopen.2023.49457)
Supplement: Supplement 2. — Data Sharing Statement [file jamanetwopen-e2349457-s002.pdf]

Data Sharing Statement

Bellerose. Differences in Self-Reported and Billed Postpartum Visits Among Medicaid-Insured Women. *JAMA Netw Open*. Published online December 27, 2023. doi:10.1001/jamanetworkopen.2023.49457

Data
Data available: No

Additional Information
Explanation for why data not available: While Pregnancy Risk Assessment Monitoring System (PRAMS) data are publicly available from the Centers for Disease Control and Prevention (CDC), we are not able to share the Medicaid enrollment records and claims data used for this analysis.
